# Socio-economic impacts of energy access through off-grid systems in rural communities: a case study of southwest Nigeria

**DOI:** 10.1098/rsta.2021.0140

**Published:** 2022-04-18

**Authors:** Samuel O. Babalola, Michael O. Daramola, Samuel A. Iwarere

**Affiliations:** Department of Chemical Engineering, Faculty of Engineering, Built Environment and Information Technology, University of Pretoria, Hatfield, Pretoria 0002, South Africa

**Keywords:** rural electrification, mini-grid, micro- and small enterprises, sustainability

## Abstract

The development of resilient energy systems is important for sustainable cities and communities. However, in countries with insufficient national energy supply, electricity distributors rarely consider remote communities due to their distant settlement, low electricity demand and poor payment capabilities. The United Nations has set a goal to deliver universal energy access by 2030; hence, it has become imperative to deploy clean and affordable off-grid mini-grid solutions to previously abandoned communities. Access to energy in rural communities is expected to result in unlocking their economic potentials. This paper investigates the impact of a solar hybrid mini-grid on the socio-economic growth of local entrepreneurs in Gbamu Gbamu village, Nigeria. A total of 83 micro- and small-enterprises has been surveyed; descriptive statistics, paired-sample *t*-test, cross-tabulation and χ^2^ test, were used to assess the performance of businesses before and after electrification. The outcomes include the number of business enterprises created, employment statistics, energy expenses and income generated. Regression analysis was conducted on the relationship between the average income generated by businesses and independent socio-economic variables such as gender, marital status, household size, age, education level, years of business establishment, hours of operation, building tenure, capital source, number of employees, generator ownership and the days of operation.

This article is part of the theme issue 'Developing resilient energy systems'.

## 1. Introduction

In the last decades, the challenge of climate change and its direct effect, global warming, has received much attention. In parallel are discussions on the management of the competing demands of the energy trilemma that are requisites for a prosperous economy [1]. As the population in developing countries continue to rise, there is an increased demand for more resilient energy systems to augment the national energy supply. Without adequate access to energy, a country may not develop beyond survival level. In this context, ‘survival level’ is defined as a condition in which the citizens of a country live under adverse and unusual circumstances such as inadequate access to other basic human needs. Despite committing a sum of USD 22.2 billion between 2009 and 2013 to improve electricity access [2], the 2020 country-by-country assessment report on global electrification by International Energy Agency [3] shows that there are still about 770 million people around the world without electricity. It was also mentioned in [3] that an average annual investment of USD 35 billion is required from 2021 to 2030 to cover the huge global electricity deficit and meet the United Nations' agenda 2030.

**Figure 1.**
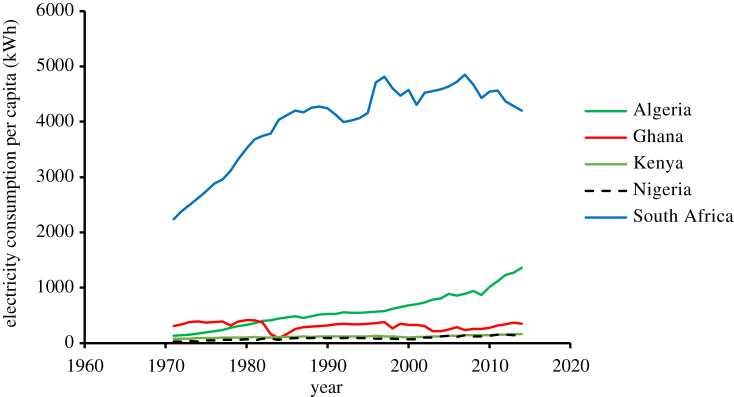
Energy consumption per capita for selected African economic giants [25]. (Online version in colour.)

For several years, Nigeria has struggled with inadequate national grid coverage and poor electricity supply. The power industry in Nigeria is built around fossil-fuel power plants that constitute about 84% of the total installed capacity. Despite having a generation capacity of about 12 522 MW, Nigeria can only boast of transmitting about 3879 MW, which is insufficient for the entire population [4]. Also, power distribution in the country favours urban settlements more than rural areas due to their huge demand and ability to pay for electricity. About 38% of the Nigerian population has no access to the national grid, while only 30% of the rural populace have access to electricity [5]. In electrified places, the quality and reliability of electricity delivered is poor. Nigeria also ranks very low in terms of electricity consumption per capita despite being an oil-producing country with huge energy resources. Figure 1 shows the electricity consumption per capita trend for selected African economic giants.

The traditional method for supplying electricity in remote areas is grid extension. However, this has often been an unattractive investment for the electricity distribution companies who need to cover an uneasy geographical terrain and a dispersed settlement. These, coupled with the low electricity demand and poor payment capabilities of the rural populace makes it uneconomical to extend the grid to some communities. As a result, the affected rural residents have been left to self-generate energy from unclean and health-threatening sources such as kerosene lamps, candles and standalone small petrol or diesel generators. Kerosene is the most prevalent lightning fuel in unelectrified villages. This is mostly used by households with a better economic status, whereas candles are found in poorer households. Petrol and diesel generators are mostly used by business owners and larger households that have higher electricity demand. These dominate both off-grid and on-grid applications in Nigeria despite their associated environmental challenges and high operating cost. In 2014, Nigerians spent a total of NGN 796 billion to fuel privately owned generators to meet the electricity deficit from the grid, which is by far the highest in the world [6].

**Figure 2.**
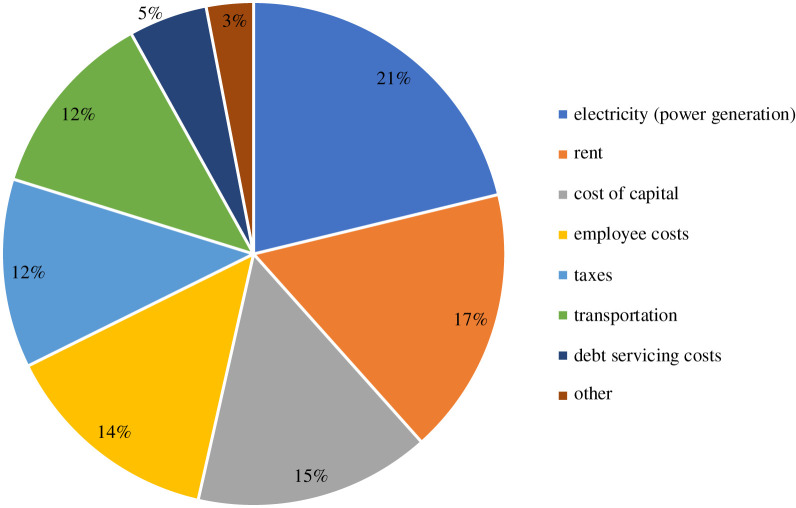
Distribution of cost incurred by micro- and small enterprises in Nigeria [26]. (Online version in colour.)

Meanwhile, Nigeria is endowed with a vast amount of renewable energy resources like biomass, solar energy, wind and hydro (pico) energy which can be harnessed for electric power generation. According to Iwayemi [5], Nigeria receives 3.5–7.0 kW m^−2^ solar radiation on a daily basis, and 2.0–4.0 m s^−1^ wind speed. It also has an estimated 14 750 MW hydro potential including 144 million tonnes per year of biomass resources. Instead of extending the grid coverage to isolated communities or using standalone generators, off-grid mini-grid renewable power systems have been recommended [7]. In this context, a mini-grid can be defined as a standalone power system or group of interconnected distributed energy resources (with or without energy storage) which generates electricity that is distributed to a localized group of people to serve their energy demand. Mini-grid is not a new phenomenon, as nearly all centralized electric power systems started as isolated mini-grid before being interconnected. A mini-grid is also a potential solution for unlocking the economic potentials of rural communities and increasing the number of income-generating activities. The World Bank describes micro- and small enterprises (MSEs) as business establishments with 1–9 employees and 10–49 employees, respectively [8]. Some of these businesses are found in rural areas, and it has been established that the MSE sector is a key driver for economic growth and employment [9]. According to a report from the Small and Medium Enterprise Development Agency of Nigeria [10], a total of 36 million Nigerian MSEs contributed about 48.47% of the country's National Gross Domestic Product in 2013. This directly implies that the growth of MSEs could be an indicator of the overall performance of an economy. However, to enable significant growth in the MSE sector in Nigeria, there are certain factors that must be addressed. Of these, power supply stands out as a priority as shown in figure 2.

While there have been several studies on the viability and challenges of mini-grids for rural communities [11] and [12], reports from academic literature have so far been inconclusive about the impact of electricity on microenterprises [13]. Thus, this study aims to contribute to the discourse in this area as a step towards closing the existing knowledge gap. The focus of this study was to show the impact of an off-grid power system on the socio-economic development of small-scale businesses in rural communities. The key questions posed and answered in this study include:

1. What are the impacts of access to electricity on rural MSEs in Nigeria, as measured by the number of new business enterprises, number of employees, petrol or diesel generator usage, the amount spent on energy before and after mini-grid connection, and income generated before and after mini-grid connection?
2. What are the factors that influence income generated by rural MSEs?

## 2. Studies on mini-grid

The concept of off-grid renewable power generation in remote areas is not uncommon. There are some studies that reported the techno-economic feasibility of mini-grid solutions in unserved communities. For example, Okoye & Oranekwu-Okoye [14] compared the levelized cost of electricity (LCOE) from solar photovoltaic (PV) to a standalone diesel generator for a remote village in the Northern Nigeria. With a USD 0.22/kWh LCOE difference in the energy technologies, the authors showed that it was cheaper to consider a solar mini-grid for power generation. In another study, Olatomiwa *et al*. [15] compared the economic implications of deploying a Solar PV/diesel/battery hybrid energy system in the six geopolitical zones of Nigeria. In this study, a diesel generator was used as a secondary source of energy and was meant to run only for a few hours. Some authors examined the possibility of having an entirely renewable energy-based mini-grid solution. A typical case was a study conducted by Bertheau [16] using a solar/wind/battery system for the electrification of an island in the Philippines. Krishan & Suhag [17] also considered three different hybrid configurations for an energy-starved community in India, and reported that a wind/solar/battery based system was the most cost-effective solution for the location considered. However, the application of wind technology in a mini-grid system is location dependent. For example, the possibility of adopting a solar/wind/diesel/battery hybrid system was investigated by Salisu *et al*. [18] for Giri village in Nigeria. In this study, the limitation of wind speed in the area and the high cost of the system showed that it would be cheaper to adopt a solar/diesel/battery system for the village instead. Some studies focused on the environmental impact of a hybrid mini-grid technology. For example, Aberilla *et al*. [19] evaluated the environmental sustainability of 21 system configurations for off-grid rural electrification in the Philippines. Based on life cycle assessment, the results from this study showed that a solar/wind/lithium-ion batteries energy system would be the most environmentally sustainable configuration for the area. Also, to show that a renewable power mini-grid is cheaper than extending the grid, some studies went further to report the breakeven grid extension distance (BGED). For instance, in South Africa, Luta & Raji [20] compared the cost of extending the grid to a community with the deployment of an off-grid system comprised solar/wind/hydrogen storage. The authors reported that it would be economically viable to implement the renewable off-grid system if the distance of the rural area to the grid was between 4728 and 8183.09 km. The results from this study suggest that grid extension might be the cheaper option in this case. Conversely, Li *et al.* [21] reported a BGED of 9.71 km for a remote village in West China which can be potentially powered by biomass, wind and solar energy resources. As a rule of thumb, Juanpera *et al.* [22] noted that extending the grid to a remote community in Nigeria is only feasible when the community is large and at a distance not longer than 25 km to the national grid.

## 3. Methodology

### (a) Brief description of project and study area

In 2015, the Nigerian Energy Support Programme supported by the German government in partnership with the Nigerian Federal Ministry of Power carefully identified some remote villages for the implementation of up to 10 Rural Electrification Pilot Projects (RrE-PP). The objective of this initiative was to demonstrate the viability of decentralized mini-grids for rural electrification in Nigeria, and also build a business case for mini-grid development in the country. Each village stood at a distance of at least 12 km from the closest grid infrastructure, thus making it difficult for a possible grid extension in the shortest years possible. The villages selected are located in geopolitical zones such as South-West, North-Central, South-South and North-West. In the South-Western zone, Gbamu-Gbamu village was selected.

**Figure 3.**
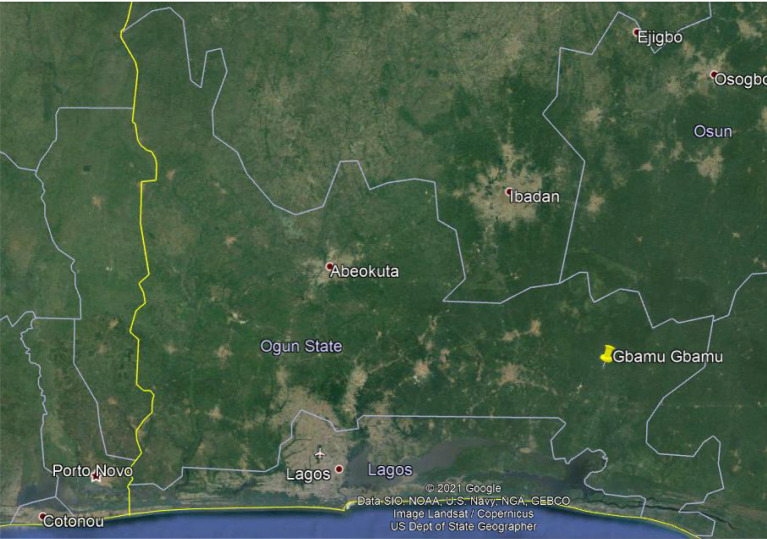
Google map showing the location of the case study. (Online version in colour.)

Gbamu-Gbamu is an agrarian community located in Ijebu East Local Government Area, Ogun East Senatorial District in Ogun State, Nigeria. This community has a population of over 3000 people (based on 2006 Census) and stands at about 130 km from the Ogun capital. The village is surrounded by forest and plantations and its distance from the closest grid infrastructure is about 13 km. The community is located on latitude 6.845387^°^N and longitude 4.214628^°^W as shown in figure 3. In addition to farming occupations, some individuals have small business establishments. In 2016, a prefeasibility assessment was conducted in Gbamu-Gbamu which took into consideration the commercial activities in the village and their business performance. This was documented by the project developer, Rubitec Solar Nigeria. Also, before the deployment of the project, an energy resource assessment was conducted in the area and this confirmed its suitability for a solar hybrid system. Gbamu-Gbamu receives a daily average solar irradiation of about 4.23 kWh m^−2^. Biomass resources are also available in the village in the form of agricultural wastes and forest trees, but considering its associated challenges such as land degradation, deforestation, pollution and the complexity of biomass technology, a solar PV technology was preferred. Consequently, an 85 kW solar/diesel hybrid mini-grid system was deployed and this serves the entire community on a 24/7 basis. The villagers buy electricity at a government-regulated tariff of about NGN 175 (approx. USD 0.48). This mini-grid project was commissioned in 2018 and is one of the first mini-grid projects to be developed in Nigeria.

### (b) Data collection and analysis

This study investigated the impact of mini-grid on business activities by comparing the socio-economic situation of MSEs in the village before and after the electric power system was developed. Owing to the unavailability of an adequate non-beneficiary population size, a pre and post treatment method has been used in this present study. The instrument used for data collection was a questionnaire with carefully and scientifically formulated questions on: social information of business owners, income-generating activities in the village, income generated by the business owners, energy expenses, and pattern of usage of petrol or diesel generator for businesses. The pre-treatment survey was conducted in 2016, while the post-treatment survey was conducted in 2021. The questionnaires were administered by a face-to-face method to 83 business owners, representing 87% of the total businesses available in the village. During the survey, the field enumerators reported the following observations:

— Some businessowners, especially the petty traders, were unwilling to participate in the study because they felt the information may be used as a political campaign.
— Other business owners may have reduced their actual income and potentially inflated their energy expenses as they believe that this could help them get financial support.

The results from the field survey were analysed with Statistical Package for Social Sciences (SPSS) v. 23. Statistical tests were also conducted to compare the relationship between the sampled variables.

### (c) Analysis techniques

#### (i) Paired (two-sample) *t*-test

The paired sample *t*-test is a statistical method that is implemented by first setting two hypotheses for the mean observations (μ_1_) and (μ_2_).

H_0_ (null hypothesis): Assumes that the true mean difference (*D*) is zero
H_1_ (alternative hypothesis): Assumes that the true mean difference (*D*) is not zero
  Thus,
  3.1 $${\text{H}}_{0}:\mu_{1}=\mu_{2}$$
  and
  3.2 $${\text{H}}_{1}:\mu_{1}\neq \mu_{2}.$$
  The paired sample *t*-test can be calculated using equation (3.3)
  3.3 $$t=\frac{D}{\sqrt{s^{2}/n}},$$
  where *D* represents the mean difference between two samples, *s*^2^ is the sample variance and *n* is the sample size.

#### (ii) χ^2^ test of independence

The Pearson χ^2^ statistical test uses a contingency table (otherwise called, cross-tabulation table) in an arrangement where the data are classified based on two categories to determine whether there is a significant difference between the expected values and the observed values in a distribution between two variables. The category for one variable appears in columns where the other variable appears in rows. Also, each variable must have two or more categories.

The general hypotheses for the χ^2^ test of association according to [23] are given below:

H_0_ (null hypothesis): Assumes that there is no significant relationship between two variables compared.

H_1_ (alternative hypothesis): Assumes that there is a significant relationship between two variables compared.

The equation used to verify the χ^2^ test of independence χ^2^ is given in equation (3.4)

3.4 $$\chi^{2}=\overset{r}{\underset{i}{\sum }}\overset{c}{\underset{j}{\sum }}\frac{{\left(n_{ij}-E_{ij}\right)}^{2}}{E_{ij}},$$

where *r* represents the number of rows; *c* represents the number of columns; *n_ij_* represents the observed value; *E_ij_* represents the expected value.

The expected value *E_ij_* is also obtained by equation (3.5)

3.5 $$E_{ij}=\frac{n_{i}\times n_{j}}{n},$$

where *n* is the number of instances, and degrees of freedom, d.f. = (*r* − 1)(*c* − 1)

#### (iii) Regression analysis

Regression analysis is a statistical method that shows how the value of the dependent variable changes with an independent variable (otherwise called predictors), while the other independent variables are held constant. In other words, with regression analysis, it is possible to estimate the conditional expectation of the dependent variable given the independent variables. Thus, it provides a better way to understand the specific independent variables which affect the dependent variables, and the forms of the relationships [24]. In this study, the relationship between the income of business owners was measured with certain variables as presented in table 1. The multiple regression model is provided in equations (3.7) and (3.8)

3.7 $$Y=\beta_{0}+\overset{n}{\underset{i}{\sum }}\beta_{i}X_{i}+e_{i}$$

and

3.8 $$Y=\beta_{0}+\beta_{1}X_{1}+\beta_{2}X_{2}+\beta_{3}X_{3}+\beta_{4}X_{4}+\beta_{5}X_{5}+\ldots +\beta_{n}X_{n}+e_{i}$$

where *Y* represents the dependent variable, *β*_0_ represents the intercept, *β*_1_ *to β_n_* represents the regression parameters or coefficients, *X*_1_ *to X_n_* represents the independent variables, *e_i_* represents the error term.

**Table 1.** Base-line information for the microentrepreneurs.

		frequency	percentage (%)
gender	male	38	45.8
	female	45	54.2
marital status	single	11	13.3
	married	71	85.5
	widowed	1	1.2
level of education	primary	1	1.2
	secondary	78	94.0
	tertiary	4	4.8
tenure of building	owned	49	59.0
	rented	34	41.0
source of capital	private fund	72	86.7
	family/friends	11	13.3
beneficiaries of tariff discount	yes	82	98.8
	no	1	1.2
type of financial institution	bank	4	4.8
	local cooperative	78	95.2
	none	1	1.2
days of business operation	less than a week	4	4.8
	throughout the week	79	95.2
number of employees employed in range	<5 employees	67	80.7
	6–10 employees	13	15.7
	>10 employees	3	3.6

## 4. Results and discussion

### (a) Socio-economic characteristics

**Figure 4.**
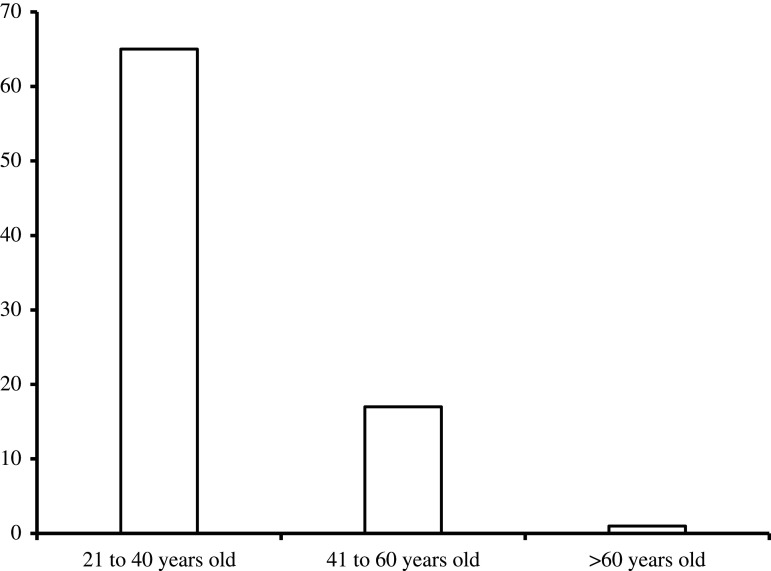
Age distribution of business owners. (Online version in colour.)

An overview of the socio-economic status of the respondents is presented in tables 1 and 2. The distribution of the respondents based on gender shows that there are higher female entrepreneurs (54.2%) than males (45.8%). The average family size of the respondents is 4. In terms of marital status, majority of the respondents (85.5%) are married, while 13.3% and 1.2% are singles and widowed, respectively. There is a significant level of literacy among the entrepreneurs as 94% of the respondents have had the basic secondary level education. Also, 4.8% have tertiary education and 1.2% are primary school leavers. Based on source of capital, 86.7% of the businesses were started with private funds while family and friends supported the remaining 13.3% with funds. According to figure 4, most of the business owners (78.3%) are within the age bracket of 21–40 years and their mean age is 35 years.

**Table 2.** Descriptive statistics.

	*N*	minimum	maximum	mean	s.d.
household size of mini-grid users	83	1	10	4.36	2.052
age of mini-grid users	83	20	70	35.59	9.143
years of business experience of mini-grid users	83	0	20	5.49	3.670
hours of operation of business	83	4.0	17.0	13.331	2.931
number of employees employed by mini-grid users	83	0	15	3.60	2.585
number of days business is in operation	83	5	7	6.94	0.286
number of months of best sales	83	1	12	5.45	2.286
maximum income generated as a beneficiary of mini-grid project per month	83	12 000	1 000 000	131 807.23	158 252.535
minimum income generated as a beneficiary of mini-grid project per month	83	4000	600 000	82 891.57	96 534.211
income generated previously as a non-beneficiary of mini-grid project per month	83	4000	400 000	59 903.01	80 968.721
amount spent on energy per month before the adoption of mini-grid project	83	800	60 000	7352.77	7243.234
amount spent on energy per month after the adoption of mini-grid project	83	800	12 000	3641.45	2559.645

Majority of the businesses surveyed in the village operate in the service sector, while others belong to the small industries and agricultural sectors. The business activities that have high electricity demand include welding, vulcanizing (air compressor), grinding, milling, cold room operation, coca drying, hotel, restaurants and commercial electric motorcycle service. These are generally referred to as productive electricity consumers as they make up a huge part of the overall electricity consumption. Also, almost all the businesses run throughout the week. A vast majority of these respondents (98.8%) enjoy a day-time tariff discount of up to 65% from 9.00 to 16.00 hours. Despite the higher electricity tariff levied on businesses operating above 16.00 hours, most of the respondents run their businesses from 8.00 to 22.00 hours. Also, almost all the businesses (95.2%) operate throughout the week. The maximum monthly energy expense incurred before and after mini-grid deployment are NGN 60 000 naira and NGN 12 000, respectively.

### (b) Impact of mini-grid

#### (i) Number and types of businesses

**Figure 5.**
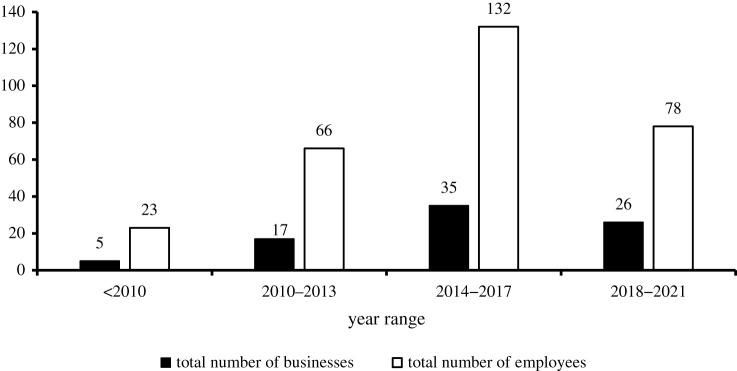
Total number of businesses opened and total employees employed. (Online version in colour.)

**Figure 6.**
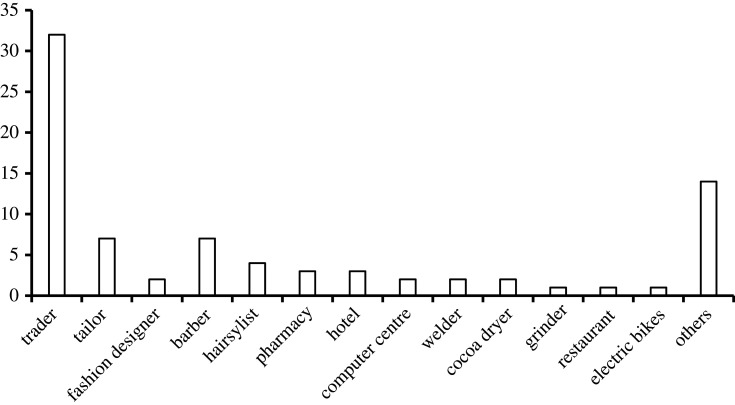
Types of businesses. (Online version in colour.)

The evolution of businesses over the last decade is shown in figure 5, while the types of businesses in the community are shown in figure 6. Among the respondents, there were only five entrepreneurs 10 years ago and these were small traders. At the end of 2013, there were 17 new businesses (triple of the previous) added to the list and almost half of these used a small fridge that was powered by a small petrol generator. Between 2014 and 2017, the number of new businesses doubled what was recorded in 2013. The trend of growth of new businesses at least doubled until the mini-grid arrived in 2018. From 2018 until 2021, the new businesses recorded is a 25.7% reduction from the previous range. With this observation, it is difficult to attribute the establishment of the 26 new businesses to the arrival of the mini-grids, as there could have been other factors responsible for this. However, it is important to note that the presence of the mini-grid has attracted innovative businesses. One of these businesses is the first-ever commercial electric motorcycles in Nigeria. This electric vehicle (EV) business officially commenced in 2020 and the owners, Max.ng, mentioned that the attraction for them was the presence of the clean electricity from the mini-grid. Further details on this project are available via the weblink: https://thenationonlineng.net/nigeria-gets-first-electric-motorcycles/. The presence of these electric motorcycles in the village has helped to reduce the use of petrol-powered motorcycles, and consequently contributed to the reduction of the carbon footprint in the village.

#### (ii) Employment opportunity

Entrepreneurs are able to create opportunities for themselves and for others as well. The total number of employees employed by microentrepreneurs have increased over a decade based on the results in figure 5. The highest average employee (3.7) was recorded between 2010 and 2013. Although as at when this study was conducted, the average employee recorded between 2018 and 2021 was the lowest (3), this figure is expected to significantly increase before the end of 2021 because there are ongoing construction projects such as a water production factory, cold rooms and more electric motorcycles will be deployed following the successful completion of the EV pilot study.

#### (iii) Comparing petrol or diesel generator usage before and after mini-grid connection

The cross-tabulation result provided in table 3, shows that a vast majority of business owners (73.7%) who previously used petrol or diesel generators had stopped using it after the mini-grid was installed in the village. The remaining 26.3% only had generators as back-ups in case of mini-grid failure. Also, 91.1% of the businesses who never depended on a generator system prior to mini-grid installation never thought about buying it after the mini-grid was installed. This is an indicator that there has been a significant reduction in the use of standalone petrol or diesel generator in the village as a result of the mini-grid, thus reducing the overall carbon footprint and other associated environmental pollution. The reason for this may be that the cost of generating electricity from standalone generators is higher than the tariff from the mini-grid. Another factor could be that the mini-grid power is much more reliable to sustain businesses.

**Table 3.** Cross-tabulation result for generator usage.

			do mini-grid users own generators previously?		
			yes	no	total
do mini-grid users own generators presently?	yes	count	10	4	14
		% within do mini-grid users own generator previously?	26.3%	8.9%	16.9%
		% of total	12.0%	4.8%	16.9%
	no	count	28	41	69
		% within do mini-grid users own generator previously?	73.7%	91.1%	83.1%
		% of total	33.7%	49.4%	83.1%
total		count	38	45	83
		% within do mini-grid users own generator previously?	100.0%	100.0%	100.0%
		% of total	45.8%	54.2%	100.0%

χ^2^ test of independence was conducted on the observation and expectation variables as shown in table 4. This requires that the variables of each cell have a frequency greater or equal to 5. The footnote ‘a' underneath the χ^2^ test box confirms this in our study indicating that our assumptions were met. The value of the test statistic is 4.462. The row of interest is the Pearson χ^2^, from which a *p*-value of 0.035 was obtained. Since the *p*-value is less than our chosen significance level (0.05), we reject the null hypothesis, as there is a significant association between the mini-grid users who previously owned petrol or diesel generators and the previous owners of these generators. On the other hand, we accept the alternative hypothesis in this case as {*X^2^* (1) = 4.462, *p* = 0.035}.

**Table 4.** χ^2^ test.

	value	d.f.	(*p*-value) asymptotic significance (2-sided)	exact sig. (2-sided)	exact sig. (1-sided)
Pearson χ^2^	4.462^a^	1	0.035		
continuity correction^b^	3.306	1	0.069		
likelihood ratio	4.529	1	0.033		
Fisher's exact test				0.043	0.034
linear-by-linear association	4.408	1	0.036		
no of valid cases	83				

^a^0 cells (0.0%) have expected count less than 5. The minimum expected count is 6.41.

^b^Computed only for a 2 × 2 table.

#### (iv) Comparing amount spent on energy before and after mini-grid connection

The comparison between the respondents' average energy expenses before and after mini-grid connection is presented in table 5. Before becoming mini-grid consumers, the microentrepreneurs spent an average of NGN 7352.77 on self-generated energy. This amount reduced by a half (50%) upon mini-grid connection, indicating that there is a non-zero true mean difference between the mean amount spent on energy before and after. The relationship between these variables was further investigated by using the paired samples *t*-test. The result of this test showed a *p*-value of 0.000, indicating that the null hypothesis can be rejected as there is a level of significance of 1% between the samples. One factor that could have contributed to the reduced energy expense is that most of the electricity users enjoy discounted tariffs during the day.

**Table 5.** Comparison for the amount spent on energy before and after mini-grid connection.

paired samples statistics									
		mean	*N*	s.d.	s.e. mean				
pair 1	amount spent on energy per month before the adoption of mini-grid project	7352.77	83	7243.234	795.048				
	amount spent on energy per month after the adoption of mini-grid project	3641.45	83	2559.645	280.958				
paired samples test									

paired differences									
					95% confidence interval of the difference				
		mean	s.d.	s.e. mean	lower	upper	t	d.f.	Sig. (2-tailed)
pair 1	amount spent on energy per month before the adoption of mini-grid project – amount spent on energy per month after the adoption of mini-grid project	3711.325	6485.541	711.881	2295.168	5127.483	5.213	82	0.000

#### (v) Business performance in terms of income

The maximum and minimum average income generated before and after connecting to the mini-grid, are provided in tables 6 and 7, respectively. The difference between the maximum average income before and after mini-grid connection is NGN 71 904.22, while the difference between minimum average income before and after NGN 22 988.55. The results also show that the entrepreneurs have experienced at least 38.4% increase in their income since connecting to the mini-grid. A paired sample *t*-test was conducted on the income before and after the mini-grid connection as shown in the tables mentioned. The paired samples test showed that there is a significant difference between the minimum and maximum average income for the mini-grid users as the *p*-value is 0.000, showing a 1% significance level. Thus, we reject we null hypotheses in the two cases table 8.

**Table 6.** Comparison for between maximum income generated before and after mini-grid connection.

paired samples statistics									
		mean	*N*	s.d.	s.e. mean				
pair 1	maximum income generated as a beneficiary of mini-grid project per month	131 807.23	83	158 252.535	17 370.472				
	income generated previously as a non-beneficiary of mini-grid project per month	59 903.01	83	80 968.721	8887.472				
paired samples test									

paired differences									
					95% confidence interval of the difference				
		mean	s.d.	s.e. mean	lower	upper	t	d.f.	sig. (2-tailed)
pair 1	maximum income generated as a beneficiary of mini-grid project per month – income generated previously as a non-beneficiary of mini-grid project per month	71 904.217	87 544.093	9609.213	52 788.432	91 020.002	7.483	82	0.000

**Table 7.** Comparison between minimum income generated before and after mini-grid connection.

paired samples statistics									
		mean	*N*	s.d.	s.e. mean				
pair 1	minimum income generated as a beneficiary of mini-grid project per month	82 891.57	83	96 534.211	10 596.006				
	income generated previously as a non-beneficiary of mini-grid project per month	59 903.01	83	80 968.721	8887.472				
paired samples test									

paired differences									
					95% confidence interval of the difference				
		mean	s.d.	s.e. mean	lower	upper	t	d.f.	sig. (2-tailed)
pair 1	minimum income generated as a beneficiary of mini-grid project per month – income generated previously as a non-beneficiary of mini-grid project per month	22 988.554	34 635.109	3801.697	15 425.768	30 551.341	6.047	82	0.000

**Table 8.** Regression results for the factors influencing business income.

variable	coefficients	s.e.	*p*-value
gender	−49 667.84^a^	27 582.29	0.075
marital status	−36 544.6	49 920.35	0.467
household size	11 535.4^a^	6751.76	0.091
age	142.0877	2045.1	0.945
educational level	39 692.77	59 209.82	0.505
year of business establishment	8827.971^b^	3713.981	0.020
hours of operation	5495.988	5242.761	0.298
building tenure	64 660.26^b^	27 577.51	0.021
capital source	56 479.77	41 046.35	0.173
number of employees	21 691.58^c^	6496.069	0.001
owning generator previously	−61 190.64^a^	34 414.54	0.079
number of days of operation	33 912.3	67 977.11	0.619
constant	−226 682.7	143 067.6	0.118
number of observations	83		
*R*^2^	0.2697		
Prob>*F*	0.0237		

^a^10% significance level.

^b^5% significance level.

^c^1% significance level.

#### (vi) Analysis of the factors that influence the average income

Socio-economic factors influencing the average income of mini-grid productive customers were analysed using the regression model presented in equation (3.7). The socio-economic variables include gender, marital status, household size, age, education level, years of business establishment, hours of operation, building tenure, capital source, number of employees, owning generator previously and the number of days at work. Six out of the 12 predictors estimated showed significant influence on the average income of the mini-grid users. These variables include gender, household size, years of business establishment, building tenure, number of employees, and owning generator previously. The *R*^2^ value estimated by the regression was 0.2697, indicating that about 27% of the average income of the productive mini-grid consumers is explained by the explanatory variables. Gender specified as a dummy was found to have a significant influence on the income generated by businesses. The direction of the coefficient is negative indicating that females tend to generate more income than males. Household size also has a significant influence on the income generated. Thus, implying that the more the household size, the more opportunities there are to generate more income from a business. This result is similar to the ‘number of employees' variable, thus indicating that household members are potential employees. The years of business establishment is another variable that influences the income generated. The result showed a positive relationship between income and years of business, signifying that older businesses generate more revenue than newer ones. The building tenure was modelled with private ownership. This means that businesses who pay rental charges for the buildings they use tend to have lesser income. Another significant variable is the previous ownership of generators. This has a negative relationship with income and indicates that the respondents who previously used generator sets have lesser income.

## 5. Conclusion and recommendations

Access to energy in rural communities has been recognized as a key contributor to socio-economic development. However, for this to be effectively realized, the energy must be reliable, affordable and environmentally sustainable. So far, the literature reports on productive electricity consumption in rural communities have not provided enough evidence on the relationship between energy access and the growth of income-generating activities. Furthermore, there is no conclusive study on the factors that influences the income of microenterprises in rural communities. This study tries to address these gaps.

To contribute to the evidence on how electricity from mini-grid influences productive activities in remote villages, an existing village with 85 kW solar hybrid power system in Nigeria was considered. In this community, a total of 83 business owners were interviewed to obtain socio-economic information before and after the arrival of the mini-grid. The data were which included information such as; the social profile of the business owners, their business information, energy expenses and income.

Descriptive statistics and analytical methods like pair sample *t*-test, cross-tabulation and χ^2^ tests were used in analysing the results. The impact of the mini-grid was accessed by the number of new businesses opened in the village, employment statistics, amount spent on energy in running businesses and the income generated as compared to the situation before connecting to the mini-grid. From the study, it was discovered that female participation in business activities was more than males. Also, although there has been an increase in the number of new businesses in the village including average number of employees from previous years, there is still no conclusive evidence that the mini-grid has caused this. To further measure the impact of the mini-grid on businesses, the authors recommend a comparison of the results from this study with a community that has no mini-grid. However, this community must have very similar conditions to provide enough evidences on how mini-grid has impacted business growth. One of the remarkable impacts of the mini-grid is the presence of innovative businesses such as commercial electric motorcycles. Also, a vast majority of the users of mini-grid have stopped using their petrol or diesel generator systems due to the reduced expense on energy from the mini-grid electricity. This decision has consequently created an income boost for them.

A regression analysis was also conducted on the relationship between the average income generated by businesses and independent socio-economic variables such as; gender, marital status, household size, age, education level, year of business establishment, hours of operation, building tenure, capital source, number of employees, owning generator previously and the number of days at work. From these variables, female gender, household size, year of business establishment, building tenure, owning generator previously and number of employees appeared to have a significant influence on the average income of the mini-grid users.

Overall, the influence of tariff discounts has played a huge role in reducing the energy expenses incurred by business owners in the village. While most mini-grid developers may not easily agree to reduce tariffs, the Nigerian government and other power stakeholders can intervene by subsidizing the tariffs paid by rural mini-grid users considering the socio-economic benefits provided in this study. Also, with the positive correlation between female gender and income, the women in the village should be particularly supported with capital in form of cash or appliance loans to start up new business entities.

## Data Availability

The datasets used and/or analysed during this study has been published by Dryad, Dataset, https://doi.org/10.5061/dryad.gqnk98sn0.
